# Selective Pyroelectric Detection of Millimetre Waves Using Ultra-Thin Metasurface Absorbers

**DOI:** 10.1038/srep21079

**Published:** 2016-02-16

**Authors:** Sergei A. Kuznetsov, Andrey G. Paulish, Miguel Navarro-Cía, Andrey V. Arzhannikov

**Affiliations:** 1 Novosibirsk State University, Pirogova St. 2, Novosibirsk, 630090, Russian Federation; 2 Budker Institute of Nuclear Physics SB RAS, Lavrentiev Ave. 11, Novosibirsk, 630090, Russian Federation; 3 Institute of Semiconductor Physics SB RAS, Novosibirsk Branch “TDIAM”, Lavrentiev Ave. 2/1, Novosibirsk, 630090, Russian Federation; 4 School of Physics and Astronomy, University of Birmingham, Birmingham B15 2TT, UK; 5 Optical and Semiconductor Devices Group, Department of Electrical and Electronic Engineering, Imperial College London, London SW7 2BT, UK

## Abstract

Sensing infrared radiation is done inexpensively with pyroelectric detectors that generate a temporary voltage when they are heated by the incident infrared radiation. Unfortunately the performance of these detectors deteriorates for longer wavelengths, leaving the detection of, for instance, millimetre-wave radiation to expensive approaches. We propose here a simple and effective method to enhance pyroelectric detection of the millimetre-wave radiation by combining a compact commercial infrared pyro-sensor with a metasurface-enabled ultra-thin absorber, which provides spectrally- and polarization-discriminated response and is 136 times thinner than the operating wavelength. It is demonstrated that, due to the small thickness and therefore the thermal capacity of the absorber, the detector keeps the high response speed and sensitivity to millimetre waves as the original infrared pyro-sensor does against the regime of infrared detection. An in-depth electromagnetic analysis of the ultra-thin resonant absorbers along with their complex characterization by a BWO-spectroscopy technique is presented. Built upon this initial study, integrated metasurface absorber pyroelectric sensors are implemented and tested experimentally, showing high sensitivity and very fast response to millimetre-wave radiation. The proposed approach paves the way for creating highly-efficient inexpensive compact sensors for spectro-polarimetric applications in the millimetre-wave and terahertz bands.

Pyroelectric detectors are proven to be one of the most effective, compact and relatively inexpensive types of uncooled thermal sensors widely used for infrared (IR) detection^1^. Nowadays, following a fast progress in terahertz (THz) and millimetre-wave (MMW) technologies^2^,^3^,^4^,^5^, a lot of efforts are being made to improve the sensitivity of pyroelectric detection at longer wavelengths λ far beyond the IR range^5^,^6^,^7^,^8^,^9^,^10^. In particular, the short MMW and sub-MMW bands (λ ≈ 0.5–3 mm) remain highly attractive for various applications, including security imaging and surveillance, nondestructive evaluation and quality control of materials etc., due to an opportunity to combine the options of relativity high penetrability of such waves through atmosphere and different non-metallic objects versus high-frequency THz, IR and optical radiation, as well as attainability of spatial resolution of the order of several millimetres acceptable for imagining of concealed targets^2^,^3^,^4^,^11^. When deploying active systems in these tasks, mostly monochromatic and linearly polarized MMW/sub-MMW radiation is normally used for object illumination. Such radiation is to be detected by a highly sensitive, compact and reliable sensor optimized for the maximal performance at a prescribed wavelength and wave polarization. Moreover, the selective MMW/sub-MMW sensors are required when multi-spectral techniques^3^,^4^,^12^ and polarization discrimination^13^,^14^,^15^ are involved in data processing.

It should be highlighted that direct application of conventional IR pyro-sensors to the MMW/sub-MMW range faces a problem of a noticeable degradation in the detector’s sensitivity that makes them appreciably inferior to Golay cells^6^. This problem appears due to rapidly diminishing absorption of long-wave radiation in a thin pyroelectric film when the wavelength increases. Currently, noticeable technological efforts are being undertaken to create a sensitive broadband pyro-detector applicable to spectroscopy tasks that is achieved via integration of absorptive metallic, oxide or carbon nanotubes coatings with pyroelectric films^9^,^10^. On the other hand, in spectrally selective detection the detector’s sensitivity can be maximized if the radiation is absorbed by a thin metamaterial structure whose heating is further sensed with a pyroelectric. It is noteworthy that metasurface absorbers enable perfect or near-to-perfect absorption within a narrow frequency band^16^,^17^,^18^,^19^ under the condition *d*/λ ≪ 1 that makes them suitable as the radiation-sensitive elements in the selective MMW/sub-MMW bolometric detectors. Such detectors enable a relatively simple adjustment of their spectral and polarization characteristics upon fabrication through a proper modification of the metamaterial structure.

In this paper, we report on the development of a compact pyroelectric detector optimized for selective sensing of the MMW radiation within a narrow spectral band centred at 140 GHz (λ = 2.14 mm). The detector is realized by combining a commercial IR pyro-sensor with a resonant metasurface absorber attached to the pyroelectric layer. Due to the small thickness of the absorber equal to 15.7 μm and corresponding to the wavelength-to-thickness ratio around 136, we minimize its thermal capacitance and, thus, achieve the high responsivity and operation speed of the detector. As a result, the developed device appears to surpass a commercial Golay cell in similar characteristics that paves the way for realizing highly efficient compact selective sensors for the MMW/sub-MMW and THz bands, which can be produced at relatively moderate technological expenses.

## Ultra-Thin Resonant Absorbers

### Basic concept and design principles

**Figure 1:**
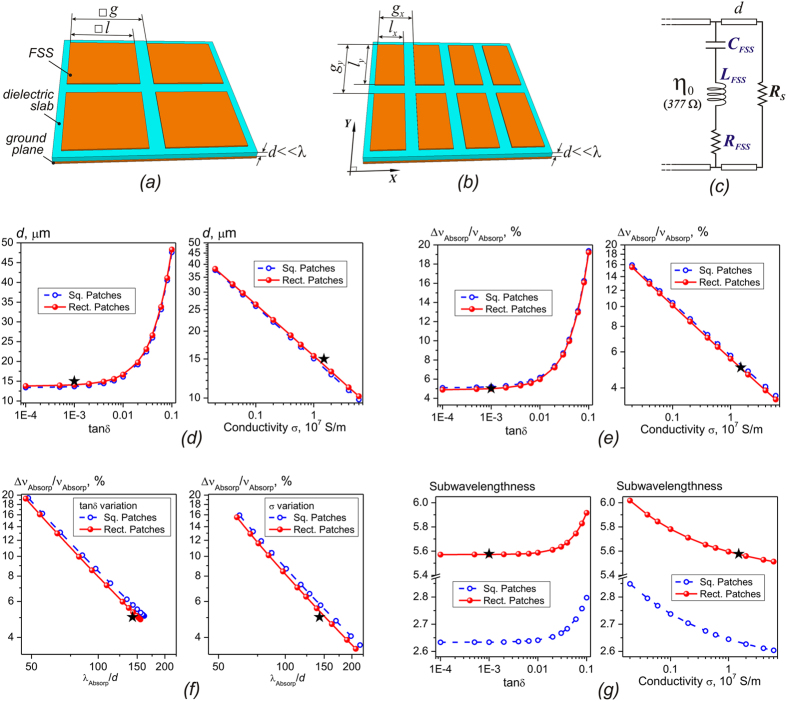
High-impedance surfaces, equivalent circuit and performance as a function of design parameters. (**a**) Square- and (**b**) rectangular-shaped metallic patch elements (**b**) frequency selective surfaces (FSS). (**c**) Equivalent circuit representation of the structures. The lossy FSS is modeled as a series connection of the lumped capacitance *C*_*FSS*_, inductance *L*_*FSS*_, and resistance *R*_*FSS*_ yielding the complex impedance *Z*_*FSS*_** = **(*j*ω*C*_*FSS*_)^−1^+*j*ω*L*_*FSS*_ + *R*_*FSS*_, which is polarization dependent in case of anisotropy. The thin dielectric backed by a lossy ground planed is described by the impedance *Z*_*S*_** = ***j*ωη_0_ *d*/*c*_0_ + *R*_*S*_. Note, a near-field “FSS – ground plane” coupling becomes considerable when *d*/λ ≪ 1 and can be formally included in {*C*_*FSS*_, *L*_*FSS*_, *R*_*FSS*_, *R*_*S*_}^25^,^32^. Tracking influence of the metallization conductivity σ and the dielectric slab loss tangent tan δ on the key parameters of perfect patch array absorbers when the FSS geometry is fixed: (**d**) thickness *d*; (**e**) fractional bandwidth; (**f**) bandwidth versus λ/*d*-ratio; (**g**) unit cell X-subwavelengthness. The unchangeable material parameters: Re(ε) = 2.25; tanδ = 1 × 10^−3^ (σ variation); σ  = 1.5 × 10^7^ S/m (tanδ variation). The star symbols indicate the actual absorber integrated with the pyroelectric sensor.

A high-impedance surface structure (HIS) combined with a lossy resistive sheet was demonstrated to be a very promising absorbing screen with deep subwavelength thickness^20^. In a basic design, the HIS is realized as a single-layer frequency-selective surface (FSS) or metasurface of a capacitive type placed above a thin grounded dielectric slab (Fig. 1(a,b))^21^. At the HIS resonance frequency, the surface impedance *Z* of the structure tends to infinity, yielding a zero reflection phase and thereby maximizing the surface electric field. The resonant E-field enhancement enables total absorption of the incident wave even without introducing an auxiliary resistive sheet but due to Ohmic or dielectric losses properly incorporated into the FSS or the carrying substrate^18^,^19^,^22^,^23^,^24^,^25^.

In an equivalent circuit formulation, the HIS-based structure is considered as a parallel connection of the FSS impedance *Z*_*FSS*_ and the impedance *Z*_*S*_of the grounded dielectric layer (Fig. 1(c)). The FSS is modelled as a series *LCR-*circuit wherein the lumped resistance *R*_*FSS*_ describes cumulative dissipative losses in FSS metallization and surrounding dielectrics^18^,^19^,^23^,^24^. In a conventional approach, the ground plane is assumed to be perfectly conductive that allows representing *Z*_*S*_in a purely inductive form when the structure is thin (*d*/λ ≪ 1): *Z*_*S*_ = *j*ω*L*_*S*_, where *L*_*S*_ = η_0_ *d*/*c*_0_, *c*_0_ is the speed of light, ω = 2π*c*_0_/λ. Actually, the Ohmic losses in the ground plane metallization can be significant for MMW/sub-MMW bands that can be taken into account by including the nonzero resistance parameter *R*_*S*_: *Z*_*S*_ = *j*ω*L*_*S*_ + *R*_*S*_. The condition of perfect absorption is achieved when the input impedance of the surface structure is matched to that of free space (see Fig. 1(c)).”

In the lossless regime (*R*_*FSS*_ + *R*_*S*_ = 0), the HIS resonance is achieved at the frequency^21^


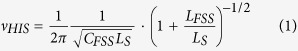


which satisfies the condition 2πν_*HIS*_·*L*_*S*_ ≪ η_0_ and corresponds to the “ultra-thinness” criterion


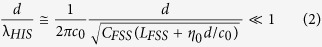


relevant for bolometers.

In our consideration it is also essential that the fractional bandwidth of the HIS resonance is bound to the *d*/λ_*HIS*_ ratio via a weakly non-linear law


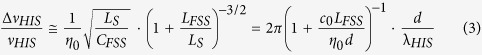


The relation in equation (3) illustrates the key feature of the HIS configuration promising for selective sensors, namely, narrowing the operational bandwidth when thinning the structure. A supplementary conclusion arising from equation (3) is that the bandwidth can be additionally decreased by augmenting the FSS inductance *L*_*FSS*_ (see also ref. 21). For instance, by comparing HIS configurations from Fig. 1(a,b) under Y-polarized excitation, one should expect the larger value of *L*_*FSS*_ and, therefore, the better spectral selectivity for the FSS with X-narrowed patch elements (b) against the square patch FSS (a).

It can be further shown that when dissipation losses are introduced into the HIS-based structure trough “switching” the resistive parameters *R*_*FSS*_ and *R*_*S*_ on, the regime of high absorptivity (i.e., when the equivalent circuit impedance matched the characteristic impedance of free space) keeps the key features of the lossless HIS. Specifically, when (*R*_*FSS*_ + *R*_*S*_)/η_0_  ≪  1 the *d*/λ-ratio of the perfect absorption, as derived from the equivalent circuit shown in Fig.1(c), is evaluated as


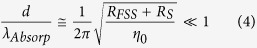


Similar to equation (3), the fractional bandwidth of the absorption resonance depends on the balance between *L*_*FSS*_ and *L*_*S*_, which is expressed by the value of 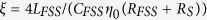 originated from the fact that *L*_*S*_now is not the independent parameter but a function of *C*_*FSS*_, *L*_*FSS*_, and (*R*_*FSS*_ + *R*_*S*_). As a result, for arbitrary ξ the bandwidth appears to relate to the thickness through a quasi-power law, which positions between the linear and quadratic dependences of the marginal cases^19^


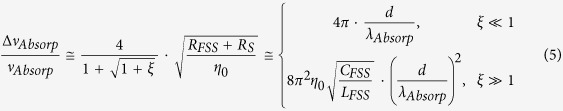


In particular, by increasing *L*_*FSS*_ and/or decreasing *C*_*FSS*_it is possible to additionally diminish the bandwidth (see also refs 19,24,25). Thus, the absorber utilizing the X-narrowed patch FSS (Fig. 1(b)) is expected to potentially provide the narrower Y-absorption resonance compared to the case of the square patch FSS (Fig. 1(a)).

It is to be highlighted that the equations (4) and (5) distinctly necessitate the employment of low-loss materials in the absorber’s configuration as it allows minimizing the thickness and bandwidth of the bolometric sensor due to decreasing the resistive parameters *R*_*FSS*_ and *R*_*S*_. By this reason, in this work we intentionally chose a polypropylene (PP) film as the low-absorbing substrate (the relative dielectric permittivity ε ≅ 2.25·(1–*j* × 10^−3^)) and the highly conductive metallization utilized in the FSS and ground layers, which was technologically implemented through sputtering aluminium. In addition, among various FSS patterns differed in a unit cell metallization filling factor, the patch elements are characterized by the smallest surface currents and, therefore, the lowest Ohmic losses^19^,^24^,^25^. As the latter ones are described via the resistance parameters *R*_*FSS*_, *R*_*S*_, the patch array FSS potentially provides the thinnest absorbing structure according to equation (4). This explains our choice in favour of the patch absorbers for the implementation of selective pyroelectric sensors reported in this work. Note, due to its relatively low permittivity ε, PP is favourable for improving spectral selectivity of the sensor as this enables to minimize the FSS capacitance *C*_*FSS*_ which governs the absorption bandwidth according to equation (5).

To validate the above-stated conclusions deduced from the analytical consideration, in Fig. 1(d–g) we present the results of examining influence of the metallization conductivity σ and the dielectric slab loss tangent tanδ on the characteristics of the fundamental absorption resonance (Y-polarized excitation, normal incidence) for perfect HIS-based absorbers utilizing patch array FSS (Fig. 1(a,b)). The absorbers with the FSS geometries described in Table 1 were numerically investigated with ANSYS HFSS™ electromagnetic software^26^ (see Methods for more details).

Anticipating the experimental results shown later, we elucidate that the geometric parameters for the absorber with rectangular-shaped patches indicated in Table 1 were found to be optimal to maximize its Y-absorptivity at 140 GHz when using the 15 μm thick PP substrate and minimizing absorption for the X-polarized wave. In this case, the following material parameters retrieved from experiments were put into simulations


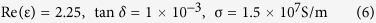


**Figure 2:**
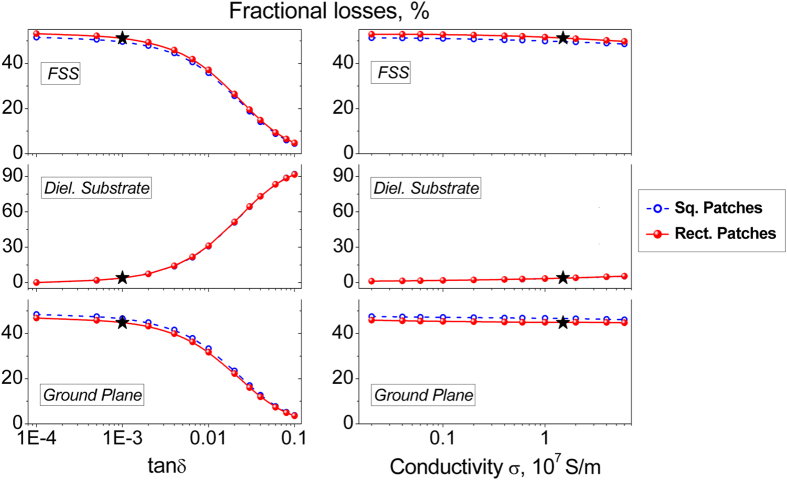
Tracking influence of the metallization conductivity σ and the dielectric slab loss tangent tanδ on the fractional dissipative losses for perfect patch array absorbers of the fixed FSS geometry. The unchangeable material parameters: Re(ε) = 2.25; tanδ = 1 × 10^−3^ (σ variation); σ = 1.5 × 10^7^ S/m (tanδ variation). The star symbols indicate the actual absorber integrated with the pyroelectric sensor.

Such an absorber marked with the star symbols in Figs 1 and 2 was chosen for the practical implementation of the pyroelectric detectors developed in this work.

Figure 1(d,e) clearly demonstrates the expected effect of gradual reduction in the absorber’s thickness *d* and the fractional bandwidth Δν_*Absorp*_/ν_*Absorp*_ when tanδ decreases or σ increases. In the latter case, both *d* and Δν_*Absorp*_/ν_*Absorp*_ values approximately behave as the 4-th root of σ indicating that the effective resistance parameter (*R*_*FSS*_ + *R*_*S*_) presented in equation (4) is near proportional to σ^−1/2^. This proves validity of using the classical expression for the surface resistance of metals to describe the resistance of the patch elements and the ground plane: (*R*_*FSS*_ + *R*_*S*_)~1/(δσ), where δ = (2/ωσ)^1/2^ is the skin depth^24^,^25^.

The data from Fig. 1(d,e) are summarized in Fig. 1(f) where the fractional bandwidth is plotted as a function of the λ/*d*-ratio. Being in concordance with equation (5), the curves are well fitted by a poorly nonlinear power dependence with a slightly varying exponent α as reported in Table 1. The presented data demonstrate feasibility to attain the λ/*d*-ratios up to 200 at the minimal bandwidth less than 4%.

By comparing graphs in Fig. 1(d–f), one can conclude that for the same material parameters both types of absorbers exhibit poor discrimination in characteristics, though the more inductive structure utilizing the X-narrowed patches (which also have the weaker inter-element capacitive coupling in Y direction) has a slightly narrower absorption band at the expense of a slight increase in its thickness, as it was predicted earlier. A striking difference is revealed only for the unit cell subwavelengthness factor *S*, which is understood herein as the ratio of the absorption wavelength to the minimal unit cell periodicity: *S* = λ_*Absorp*_/*g*_*x*_ (Fig. 2(g)). The parameter *S* is important for the practical implementation of bolometric detectors having a limited diameter of the radiation-sensitive area. Indeed, *S* defines the total number of FSS unit cells involved in the interaction with the MMW-radiation incident upon a sensitive element. If this number becomes too small, it may cause degradation in the absorber performance and, therefore, the detector responsivity. From this point of view, the geometry of rectangular patches surpasses more than twice the geometry of square patches in the *S* value (Fig. 2(g)).

We should pay attention that due to anisotropy the rectangular shape geometry provides strong polarization discrimination. For instance, under X-polarized excitation the fundamental absorption resonance of the absorber marked with the star symbols in Figs 1 and 2 falls on 327 GHz versus 140 GHz referred to Y-polarization. This makes the rectangular patch absorber ideal for realizing polarization selective pyro-sensors. All the aforementioned potential advantages of the rectangular-shaped patch array absorbers are summarized in Table 1.

In addition to the foregoing analysis, an important remark should be made concerning fractional distributions of dissipative losses in the patch array absorbing structures. According to simulations reported in Fig. 2, the Ohmic losses in the ground plane (GP) are evaluated to be at the same level as for the FSS. For the “star” absorber, the fractional losses *A* are concentrated mainly in metallization and distributed as follows:


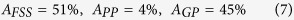


An in-depth examination of HIS-based absorbers of alternative FSS geometries (results not reported here) shows that for the much more inductive FSS (e.g. split ring resonators arrays) the losses in the FSS become strongly predominant due to a noticeable increase of the resistive parameters *R*_*FSS*_ versus *R*_*S*_. In case of patch arrays, the fact that almost a half of the radiation power is dissipated in the ground plane serves as a favorable factor for the pyroelectric sensor. Indeed, when the absorber faces the pyro-sensor from the GP side being in a close contact with it, the heat is transferred more rapidly to the pyroelectric layer that minimizes losses in the detector’s sensitivity and the response time.

### Measurements

Owing to the above discussion, the HIS-based absorbing structure utilizing the rectangular patch array FSS presented in Table 1 was chosen in this work to demonstrate efficient pyroelectric detection of 140 GHz radiation with high frequency and polarization discrimination. The thickness *d* = 15 μm for the employed PP substrate was selected among an available discrete set of thicknesses from the “GoodFellow” manufacturer^27^ as the optimal value providing maximal Y- absorptivity *A*_*y*_ and minimal X-absorptivity *A*_*x*_ at 140 GHz under conditions (6). The theoretical quantities were found to be


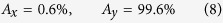


that with an accuracy 0.4% allows us considering the Y-absorptivity as perfect. In this case, the factional bandwidth for the 15 μm-PP-absorber is evaluated to be 5.0% at the λ/*d*-ratio of 142.8 (or 136.4 if calculated for the total thickness (*d* + 2*t*)=15.7 μm).

**Figure 3:**
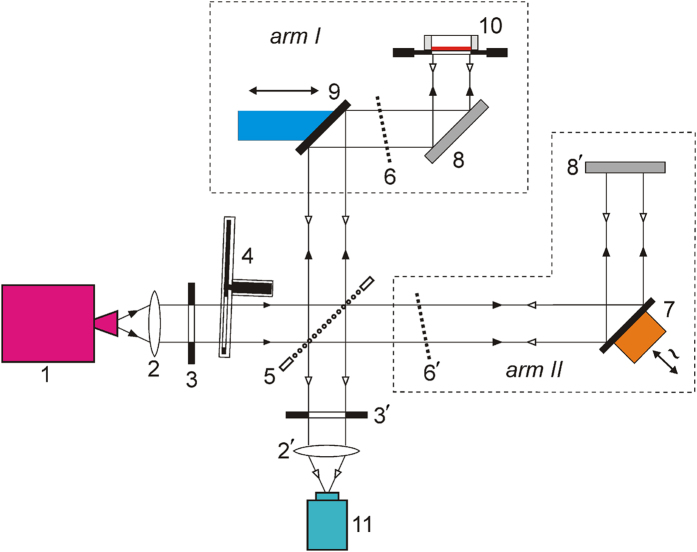
Michelson interferometer arrangement of the quasi-optical BWO-spectrometer used for measuring complex reflectivity of flat samples. 1 – radiation source (BWO); 2, 2′ – lenses; 3, 3′ – diaphragms; 4 – chopper; 5 – beam splitter; 6, 6′ – wire grid polarizers; 7 – phase modulator; 8, 8′ – fixed mirrors; 9 – movable mirror mounted on a translation stage; 10 – sample mount; 11 – detector (Golay cell). In amplitude measurements the arm II is not used.

**Figure 4:**
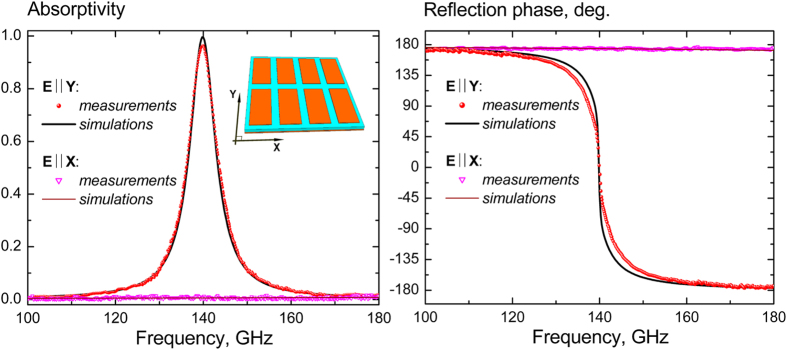
Spectral performance of the 50** **mm-CAD resonant absorber utilizing the rectangular patch array FSS and the 15 μm-PP-substrate (normal incidence illumination).

The performance of the 15 μm-PP-absorbers fabricated via photolithography^28^ and having a clear aperture diameter (CAD) of 50 mm was experimentally investigated within the spectral range of 100–180 GHz by using a CW quasi-optical backward wave oscillator (BWO) spectrometer from Microtech Instruments, Inc.^29^. To enable complex reflectivity measurements, the spectrometer was rearranged in the scheme of the polarizing Michelson interferometer shown in Fig. 3, which represents the modified scheme of ref. 30 (see Methods for more details). The typical amplitude and phase characteristics of the 50 mm-CAD absorber measured with the foregoing technique are shown in Fig. 4. The presented experimental data demonstrate good agreement with the simulation results. It is distinctively seen that for the Y-polarized excitation the reflection phase passes through zero at the frequency of the absorptivity maximum (140 GHz) as it was expected in the HIS approach. The actually measured peak magnitude of the Y-absorptivity is evaluated to be *A*_*y*_ ≅ 96.4% at the X-absorptivity mean *A*_*x*_ ≅ 0.8%. These values yield 21 dB of the polarization discrimination attainable for the developed absorber.

## Pyroelectric Detection

To demonstrate the efficient frequency- and polarization-selective detection of millimetre waves, the developed 15 μm-PP-absorber was integrated with the commercial discrete pyroelectric sensor MG33 from the Russian manufacturer “Vostok”^31^. Originally optimized for sensing IR radiation within the wavelength range of 2–20 μm, this kind of sensors exhibits the typical voltage responsivity of 10^5^ V/W at the noise equivalent power (NEP) around 1.0 × 10^−9^ W/Hz^1/2^.

**Figure 5:**
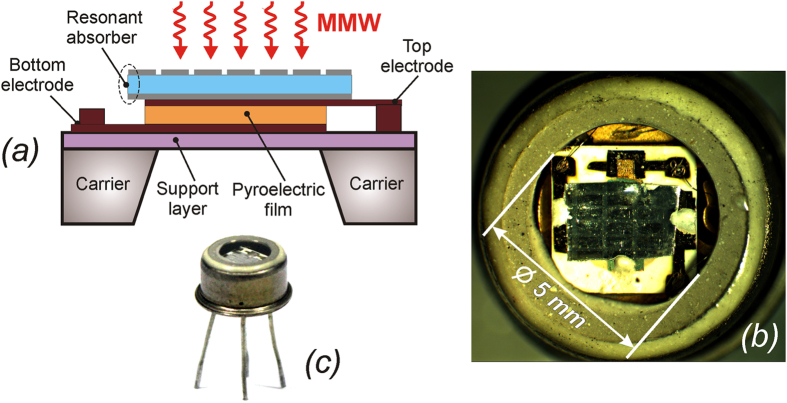
Pyroelectric sensor. (**a**) Sketch of the pyroelectric sensor with an integrated resonant absorber. (**b**) Photo of the sensor structure through the sapphire window. (**c**) Appearance of the accomplished sensor in the standard KT-3 package.

The basic scheme of the pyroelectric sensor combined with the resonant absorber is depicted in Fig. 5(a). In this work we attached the resonant absorber directly to the top electrode of the pyroelectric film and fixed it around the periphery by a heat-conducting paste. The resulting packaged structures are shown in Fig. 5(b). Further details of the integration process are described in Methods. Due to fabrication constraints, two detectors with slightly different absorbing areas were prepared for testing. The absorber’s dimensions were correspondingly chosen to be 2.47 × 2.30 mm^2^ (*Prototype #1*) and 2.47 × 1.54 mm^2^ (*Prototype #2*) that included only 3 × 6 and 3 × 4 resonant patches in the FSS pattern respectively. From the fundamental point of view, it is of great interest to ascertain the influence of such a small quantity of resonant FSS elements interacting with the incident electromagnetic radiation on the detector performance.

### Spectral and polarization selectivity

**Figure 6:**
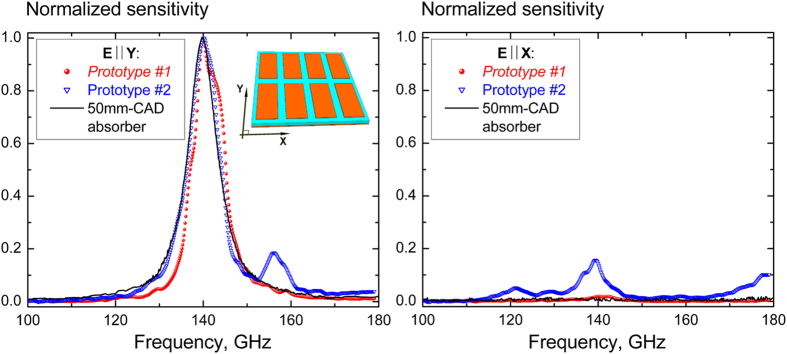
Normalized spectral sensitivity of the pyroelectric detectors differed in the operating area of the integrated resonant absorbers. The solid line indicates the normalized absorptivity of the large area absorber with the clear aperture diameter of 50 mm.

The spectral properties of the detector prototypes were examined within the frequency range of 100–180 GHz using the quasi-optical BWO-spectrometer mentioned earlier (see details in Methods). The results of spectral measurements are shown in Fig. 6, wherein the detector signal is additionally normalized to its maximum to better guide the eye when comparing the spectral bandwidth of different detector prototypes. The top and bottom graphs correspond to co-polar (**E** || Y) and cross-polar (**E** || X) illumination respectively. It can be seen that for the *Prototype #1* its spectral sensitivity correlates well with the absorptivity of the 50** **mm-CAD absorber exhibiting more than 18 dB of the polarization discrimination at 140 GHz. It is pertinent to notice that the co-polar sensitivity reveals the onset of the weak extra maximum at 142.1 GHz which slightly deforms the spectral performance of the *Prototype #1*. The similar spurious secondary co-polar peak positioned at 156.1 GHz manifests more distinctly for the *Prototype #2*, which utilizes the smaller area absorber and demonstrates the degraded polarization discrimination level (8 dB only).

The revealed effects of the fundamental absorption resonance spectral splitting and the polarization selectivity degradation can be associated with a limited number of the FSS unit cells forming the employed absorbing structures. These “area-related” phenomena cannot be predicted in the framework of the infinite array absorber model and require further research for their adequate electromagnetic description. Note that, due to technological and instrumentation constraint, the experimental characterization of the isolated absorbers in this work was limited only by the 50 mm CAD samples that made impossible the direct measurements of the absorptivity for small area structures like those integrated onto the pyroelectric sensor. At the same time, from the presented experimental data one can deduce that the integrated absorbers retain their high performance despite a relatively small number of the FSS unit cells, while covering the area within 0.72 ÷ 1.15·λ_*Absorp*_ only. This remarkable fact important for detector miniaturization is inherent in the “ultra-thinness” regime and originates from the much stronger near-field coupling between the FSS and GP layers compared to the interaction between the adjacent patch elements^25^,^32^. In terms of the equivalent circuit model, this means that the effective capacitance *C*_*FSS*_ rather becomes a function of the thickness *d* than the inter-patch gap *a*, making the absorber weakly sensitive to the number of the illuminated patch elements. Such a statement is valid when *d* ≪ *a* that is fulfilled in our case as the gap values referred to X and Y axes are equal to 103 μm and 132 μm respectively (see Table 1).

### Voltage responsivity

The voltage responsivity of the detector prototypes was measured using the absolute THz power meter from TK Instruments, Ltd.^33^. Due to its large radiation-sensitive area, this device is optimal for determining power of free-space beams with a diameter up to 30 mm.

In our experiments with CW illumination from the BWO source, the total mean power for the MMW flux focused onto the input window of the pyro-detector at the frequency of 140 GHz and 23** **Hz-modulation optimal for the power meter operation was found to be 2.6 mW. It was further taken into consideration that only a fraction of the incident energy is registered by the detector due to the limited area of the absorber. The fraction value was correctly estimated from the spatial distribution of the MMW beam intensity fallen on the absorbing area, which was determined experimentally through 2D mechanical scanning of the detector in the plane of its input window. It was also taken into account that ultra-thin metamaterial absorbers exhibit high angular stability of their performance under oblique incidence (up to several tens of degrees at least, see e.g. refs 16,17,23,25) that allowed us to neglect changes in the absorptivity when irradiating the pyro-detector with a converging beam formed by a focusing lens. As a result, at the fundamental absorption frequency of 140 GHz, the fractions of the incoming energy flux registered by the *Prototype #1* and *Prototype #2* were evaluated as 46% and 33% respectively. Measured at 23 Hz of the modulation frequency, the resulted voltage responsivities of the detectors were found to be 56 kV/W for the *Prototype #1* and 40 kV/W for the *Prototype #2*, while their NEP was estimated as 2.0 × 10^−9^ W/Hz^1/2^. It is noteworthy that, when examined with the same detection system, the IR responsivity and NEP values of the original MG33 detector before its integration with the resonant absorber were estimated to be 90–110 kV/W and 1.0 × 10^−9^ W/Hz^1/2^ respectively. Thus, the presence of the 15 μm-PP-absorber does not lead to a noticeable deterioration of the detector performance in the MMW regime, which is characterized by the degradation factor of 2.

The magnitudes of the output signals from the pyroelectric detectors were also compared with the similar response of the Golay cell from the BWO-spectrometer kit^34^ using the same lock-in detection system. A difference in dimensions of the radiation-sensitive areas of the detectors was taken into account. As a result, at the chopper frequency of 23 Hz the pyroelectric signals generated under 140** **GHz-illumination were found to exceed the Golay cell signals by the factors of 3.8 and 3.0 referred to the *Prototype #1* and the *Prototype #2* respectively.

### Response time

**Figure 7:**
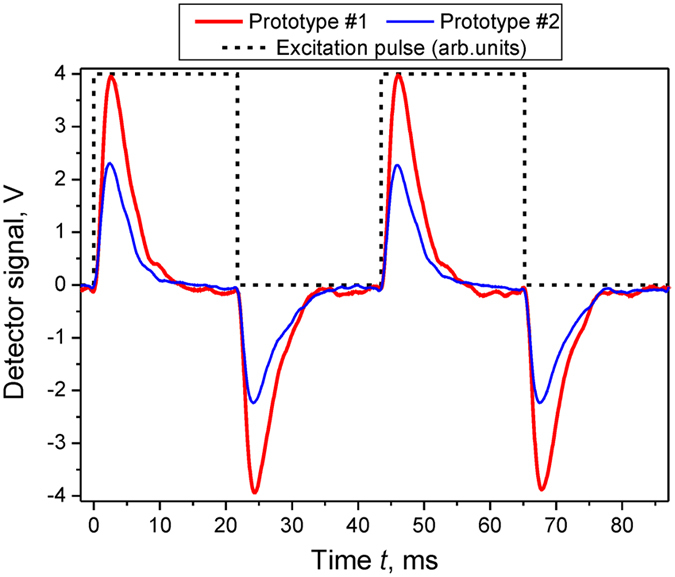
Time response of the pyroelectric detectors with integrated resonant absorbers under meander-pulsed excitation.

The response time of the pyroelectric detectors was identified via inspecting the time behaviour of the their signals under CW 140** **GHz-illumination modulated by a 23** **GHz-meander-waveform pulse with the unit on-off time ratio, as shown in Fig. 7. For this purpose, a fast-speed PIN modulator attached to the output flange of the BWO providing the wavefront rise/decay time around 1 ns was used. The experimental signals from the pyro-detectors were mathematically processed by fitting the falling edges in their time characteristics with a damped exponential function *exp*(*t*/τ). The resulted response time τ was evaluated to be 2.3 ms for the *Prototype #1* and 3.0 ms for the *Prototype #2.* For comparison, the original MG33 detector free from the resonant absorber showed the larger value of τ estimated as 4.1 ms. This means that the attachment of the absorbing structure augments the thermal conductance *G* between the sensing element and its surroundings that inevitably decreases τ according to the inverse proportionality law: τ ~ 1/*G* (see ref. 1). Our thermophysical modelling shows that such an augmentation is attributed mainly to the heat transfer along the GP layer of the resonant absorber.

An additional factor favourably minimizing the integral response speed of the pyro-detector is related to the fact that a large fractional amount of the incoming MMW energy is deposited in the GP layer, as it was established in Section Ultra-thin resonant absorbers (see relations (7)). This effect, however, has a direct action mainly upon the rise-up edge of the time characteristics which depends on the heat transfer from the FSS to GP layers through the PP film. For the 15 μm thick PP substrate, the thermal diffusion time is evaluated to be 2.3 ms.

To conclude, in this work we demonstrated a relatively simple and effective approach for selective detection of millimetre waves within a narrow spectral band centred at 140 GHz by using an ultra-thin near-to-perfect resonant absorber integrated with a compact IR pyroelectric sensor. Fabricated independently as a self-supporting thin-film metamaterial structure with the wavelength-to-thickness ratio of 136.4, the absorber is realized in a high-impedance surface configuration (FSS-PP-GP) whose ground plane layer is then attached to a top electrode of the pyro-sensor. As no drastic modification of the original IR sensor is undertaken, excluding substitution of the input window material, such radiation detectors can be produced at relatively low technological expenses. By the example of the patch array absorbers, it is shown that the detector keeps its high performance even though the absorbing area is confined within λ × λ dimensions. This significant result is explained by the strong FSS-GP capacitive coupling inherent for the “ultra-thinness” regime. The proposed approach is estimated to be promising for creating effective, miniature and inexpensive pyro-detectors applicable to spectro-polarimetric measurements both in the millimetre-wave and terahertz bands. It is ideal for monitoring the radiation beams of the submilliwatt power level and higher. Such a kind of detectors can be easily integrated into 1D or 2D arrays capable of examining the radiation spectrum and polarization for the incoming beam without using supplementary dispersive elements. In this case, the operation wavelength for each discrete channel can be adjusted via a proper modification of the FSS pattern and the substrate thickness *d*.

### Methods

#### Full-wave simulation

The absorbers were numerically investigated with ANSYS HFSS™ electromagnetic software^26^ by exploiting the regime of Floquet ports and periodic boundary conditions applied to the structure unit cell. In simulations, the FSS geometry was fixed according to Table 1, while the optimal PP slab thickness *d* and the absorption frequency ν_*Absorp*_ were retrieved with ANSYS HFSS™ via minimizing the absorber reflectance (|*S*_*11*_|^2^ ≤ –60 dB). The simulations were carried out for the metallization thickness *t* = 0.35 μm imposed on the patch elements and the ground plane layer by our fabrication technology. Note, the *g* and *l* dimensions referred to the Y-axis were chosen to be identical for both types of absorbers from Table 1 to adequately compare their performance under Y-polarized excitation.

#### Fabrication

The optimal 15 μm-PP-absorbers having the clear aperture diameter (CAD) of 50 mm were fabricated using a photolithographic patterning technique described in ref. 28. Such a large aperture was intentionally chosen to minimize instrumental errors when characterizing the absorbers with a quasi-optical BWO-spectroscopy technique applied in this work, as well as to minimize a possible discrepancy between measurements and numerical simulations treating the absorbers as the infinite periodic arrays. Note, though a thermal vacuum deposition of aluminium was applied in this work as a best method of maximizing the metallization conductivity σ for the FSS and the ground plane layers, we found that the surface roughness of the PP films^28^ typically limits σ to the level indicated in (6).

#### BWO-based Michelson interferometer

The polarizing Michelson interferometer arrangement shown in Fig. 3 was employed to enable both amplitude and phase measurements of the complex reflectivity. In the presented arrangement, the coherent MMW radiation from the BWO passed through a wavefront-correcting lens 2 is emitted into free space as a diffraction-limited Gaussian beam. To realize phase-sensitive measurements, the beam is split with a wire grid beam splitter 5 onto two orthogonally-polarized MMW beams propagating in the different arms of the interferometer. The examined absorbing structure strongly tightened onto an annular metallic holder is placed in the position 10 of the *arm I* and is illuminated under normal incidence, similarly to the flat metallic mirror 8′ edging the *arm II*. The wire grid polarizers 6, 6′ are introduced into the arms having 45 deg-orientation of their wires relative to the initial polarization of the beams that enables beam interference in the focal plane of the Golay cell detector 11. During measurements, the optical lengths for both arms are adjusted to maintain a zero-order interference minimum that is realized by using a computer-controlled longitudinal translation stage combined with a mirror 9, which is placed in the *arm I* and has a positioning accuracy of 0.5 μm. In the *arm II*, the mirror 7 attached to the 23-GHz-vibrating membrane of a loudspeaker plays a role of a modulator used for the lock-in detection. In the measurement procedure, the reflection phase of the investigated sample is retrieved by a spectrometer software from a difference in the recorded positions of the mirror 9 obtained when the investigated sample is fixed in the mount 10 and when it is substituted for a plain mirror^30^. Note, *the arm II* of the interferometer is not used when measuring the reflection amplitude. In this case, the mechanical chopper 4 providing 23 GHz shuttering is employed for the amplitude modulation.

For the investigation of the integrated metasurface absorber pyroelectric sensor, the BWO-beam was focused onto the input window of the detector with the aid of a polyethylene lens from a standard kit of the spectrometer having the focal distance and CAD values of 60 mm and 50 mm respectively. To eliminate spectral features originated from non-uniform spectral behavior of the BWO emission, as well as from quasi-optical effects attributed to MMW beam propagation in the spectrometer, the radiation intensity registered by the pyroelectric detector was normalized to the signal of the Golay cell, which was placed in the same position as the pyro-detector. To control the radiation polarization, a wire grid polarizer was applied in the experiments. The polarizer provided at least 40 dB in the polarization discrimination between the radiation components polarized along and perpendicular to the wires.

#### Integrated resonant absorber pyroelectric sensor:

The basic scheme of the pyroelectric sensor combined with the resonant absorber is depicted in Fig. 5(a). The sensor includes a thin pyroelectric film with top and bottom metallic electrodes, which are connected with a preamplifier and collect the pyroelectric charge induced in the pyro-film upon its heating by the incoming radiation. The structure is formed on a supporting dielectric layer fixed on a glassceramic carrier with a through-hole in the middle to reduce heat dissipation. In this work we attached the resonant absorber directly to the top electrode of the pyroelectric film and fixed it around the periphery by a heat-conducting paste. The resulting structures were housed in the Ar-filled KT-3 package, standard for the MG33 sensor, wherein the original germanium window was substituted for a sapphire slab 350 μm thick to provide its transparency for the incoming 140 GHz radiation (Fig. 5(b)). Note, due to constructional limitations in the MG33 sensor design, the radiation sensitive area of the detector was limited by the diameter of 2.5 mm that obliged us to properly diminish the overall dimensions of the resonant absorber. As a result, the two detectors with slightly different absorbing areas were prepared for testing. The absorber’s dimensions were correspondingly chosen to be 2.47 × 2.30 mm^2^ (*Prototype #1*) and 2.47 × 1.54 mm^2^ (*Prototype #2*) that included only 3 × 6 and 3 × 4 resonant patches in the FSS pattern respectively.

## Additional Information

**How to cite this article**: Kuznetsov, S. A. *et al*. Selective Pyroelectric Detection of Millimetre Waves Using Ultra-Thin Metasurface Absorbers. *Sci. Rep.* **6**, 21079; doi: 10.1038/srep21079 (2016).

## Figures and Tables

**Table 1:**
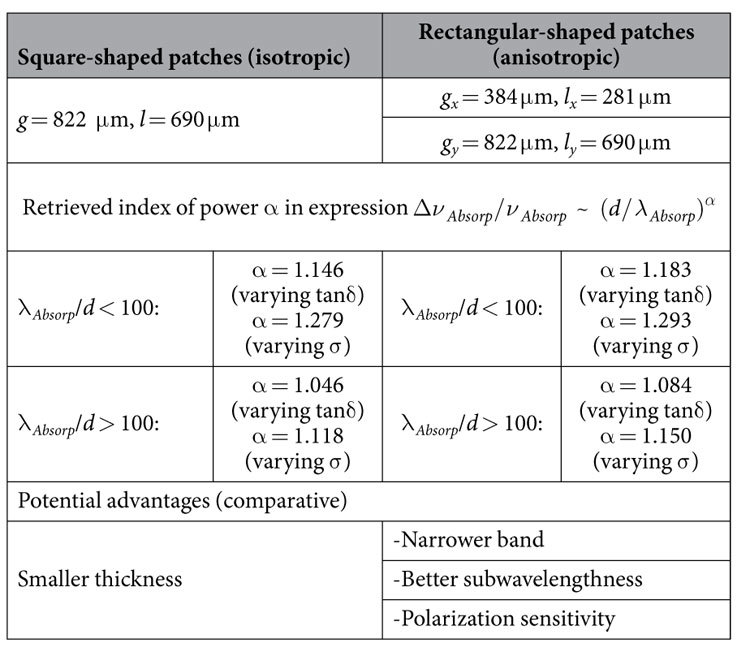
Characteristics of investigated patch array absorbers.
